# Patients Will Lie

**Published:** 1884-05

**Authors:** 


### SELECTIONS.

PATIENTS WILL LIE.—In an article on *Practical Hints*, by Dr. Landesbery, in the *Bulletin*, he speaks of how patients will occasionally undertake to deceive their physicians: "Don't trust anybody, be it monk or nun. Rely only upon the evidence of your senses. Rank, wealth, and position are only the mantle which covers the poor flesh, and you have to deal with the *materia peccans*. In this world in which we live nothing is more likely to happen than what people generally call 'the improbable' and 'impossible,' and the physician has to be on the guard against 'surprises.'"

